# Supplementary material for: Development of an improved blood-stage malaria vaccine targeting the essential RH5-CyRPA-RIPR invasion complex
Source: Nat Commun. 2024 Jun 7;15:4857. doi: 10.1038/s41467-024-48721-3 (PMC11161584; doi:10.1038/s41467-024-48721-3)
Supplement: Supplementary file 3 — Reporting Summary [file 41467_2024_48721_MOESM3_ESM.pdf]

nature portfolio

Corresponding author(s): Barnabas Williams
Simon Draper

Last updated by author(s): Apr 3, 2024

Reporting Summary

Nature Portfolio wishes to improve the reproducibility of the work that we publish. This form provides structure for consistency and transparency in reporting. For further information on Nature Portfolio policies, see our Editorial Policies and the Editorial Policy Checklist.

Statistics

For all statistical analyses, confirm that the following items are present in the figure legend, table legend, main text, or Methods section.

n/a	Confirmed	
☐	☒	The exact sample size (*n*) for each experimental group/condition, given as a discrete number and unit of measurement
☐	☒	A statement on whether measurements were taken from distinct samples or whether the same sample was measured repeatedly
☐	☒	The statistical test(s) used AND whether they are one- or two-sided<br>*Only common tests should be described solely by name; describe more complex techniques in the Methods section.*
☒	☐	A description of all covariates tested
☐	☒	A description of any assumptions or corrections, such as tests of normality and adjustment for multiple comparisons
☐	☒	A full description of the statistical parameters including central tendency (e.g. means) or other basic estimates (e.g. regression coefficient) AND variation (e.g. standard deviation) or associated estimates of uncertainty (e.g. confidence intervals)
☐	☒	For null hypothesis testing, the test statistic (e.g. *F*, *t*, *r*) with confidence intervals, effect sizes, degrees of freedom and *P* value noted<br>*Give P values as exact values whenever suitable.*
☒	☐	For Bayesian analysis, information on the choice of priors and Markov chain Monte Carlo settings
☒	☐	For hierarchical and complex designs, identification of the appropriate level for tests and full reporting of outcomes
☒	☐	Estimates of effect sizes (e.g. Cohen's *d*, Pearson's *r*), indicating how they were calculated

Our web collection on statistics for biologists contains articles on many of the points above.

Software and code

Policy information about availability of computer code

Data collection	Software used for data collection are commercially available or openly accessible. SPR data were collected using Biacore™ X100 machine and control software version 2.0.2. ELISA data were collected on Biotek ELx808, Gen5 software version 3.11. GIA data were collected on BioTek 800 TS, Gen5 version 3.11.
Data analysis	Software used for data analysis are commercially available or openly accessible as described in the methods section SPR data were analysed using Biacore™ X100 evaluation software version 2.0.2. ELISA and GIA data were analysed on Gen5 software version 3.11. Structures were visualised using UCSF ChimeraX version 1.7.1. GraphPad version 10.2.2 was used to generate graphs and for statistical analysis.

For manuscripts utilizing custom algorithms or software that are central to the research but not yet described in published literature, software must be made available to editors and reviewers. We strongly encourage code deposition in a community repository (e.g. GitHub). See the Nature Portfolio guidelines for submitting code & software for further information.

Data

Policy information about availability of data

All manuscripts must include a data availability statement. This statement should provide the following information, where applicable:

- Accession codes, unique identifiers, or web links for publicly available datasets
- A description of any restrictions on data availability
- For clinical datasets or third party data, please ensure that the statement adheres to our policy

Data are available within the Article, Supplementary Information or Source Data file. Source data are provided with this paper.
PDB structures used in this study: 4U0Q, 7PHU, 6MPV, 8CDD, 7PI3, 7P17, and 6MPV.

Research involving human participants, their data, or biological material

Policy information about studies with human participants or human data. See also policy information about sex, gender (identity/presentation), and sexual orientation and race, ethnicity and racism.

Reporting on sex and gender	N/A
Reporting on race, ethnicity, or other socially relevant groupings	N/A
Population characteristics	N/A
Recruitment	N/A
Ethics oversight	N/A

Note that full information on the approval of the study protocol must also be provided in the manuscript.

Field-specific reporting

Please select the one below that is the best fit for your research. If you are not sure, read the appropriate sections before making your selection.

☒ Life sciences ☐ Behavioural & social sciences ☐ Ecological, evolutionary & environmental sciences

For a reference copy of the document with all sections, see nature.com/documents/nr-reporting-summary-flat.pdf

Life sciences study design

All studies must disclose on these points even when the disclosure is negative.

Sample size	Sample sizes are described in figure legends. Group sizes in some rat experiments were selected based on practical considerations related to screening vaccine antigens. Some vaccine constructs were repeated with group sizes to provide sufficient power to asses statistical differences in the immune response of interest versus a gold-standard group.<br><br>Mouse experiment size (for generating monoclonal antibodies through hybridoma work) was chosen to provide sufficient material for multiple rounds of hybridoma fusion.<br><br>For rabbit experiments between 2 and 4 animals were immunised. Power calculations were not carried out since these animals were used to generate pools of anti-PfRIPR purified IgG as tools for GIA reversal studies, not for statistical comparison of different vaccine groups. Sample sizes were chosen to provide sufficient serum material for all assays conducted in this paper to reduce batch to batch variation.
Data exclusions	GIA data from immunisations with RIPR EGF (7-8) (20ug) (Figure 6 E,G) was excluded due to high growth-inhibition assay readings data but lack of ELISA response indicating a background effect.
Replication	The number of repeats for each experiment is given in the figure legends and methods. Typically, experiments were performed in independent technical triplicates.
Randomization	Animal studies were carried out by independent contractors as described in the methods. Animals were not randomised to group however all variables except immunogen were kept constant.
Blinding	GIA assays were performed blinded by two dedicated research assistants. The other investigators were not blinded to the group allocations during the experiment and analysis. Not all investigators could be blinded due to the complexity of coordinating the studies with a CMO and across experimental disciplines. Analysis was performed on quantitative endpoints to reduce investigator bias.

Reporting for specific materials, systems and methods

We require information from authors about some types of materials, experimental systems and methods used in many studies. Here, indicate whether each material, system or method listed is relevant to your study. If you are not sure if a list item applies to your research, read the appropriate section before selecting a response.

Materials & experimental systems

n/a	Involved in the study
☐	☒ Antibodies
☐	☒ Eukaryotic cell lines
☒	☐ Palaeontology and archaeology
☐	☒ Animals and other organisms
☒	☐ Clinical data
☒	☐ Dual use research of concern
☒	☐ Plants

Methods

n/a	Involved in the study
☒	☐ ChIP-seq
☒	☐ Flow cytometry
☒	☐ MRI-based neuroimaging

Antibodies

Antibodies used	Goat-anti-rat IgG (whole molecule)–Alkaline Phosphatase antibody (Sigma-Aldrich A8438; secondary antibody, lot no. SLCH0835)<br>Goat-anti-human IgG (γ-chain specific)–Alkaline Phosphatase antibody (Sigma-Aldrich A3187; secondary antibody, lot no. 0000278654)<br>Goat-anti-mouse IgG (whole molecule)–Alkaline Phosphatase antibody (Sigma-Aldrich A3562; secondary antibody, lot no. SLCP2562)<br>Goat-anti-rabbit IgG (whole molecule)–Alkaline Phosphatase antibody (Sigma-Aldrich A3812; secondary antibody, lot no. SLBR5530V)<br>Rabbit-anti-RIPR (polyclonal, primary antibody) and Rabbit-anti-CyRPA (polyclonal, primary antibody) were generated in this article.<br>Anti-RH5 antibodies R5.001, R5.002, R5.003, R5.004, R5.006, R5.007, R5.008, R5.009, R5.010, R5.011, R5.013, R5.014, R5.015, R5.015, R5.017, R5.017, R5.018, and R5.019 are as described in PMID 31204103. Anti-RH5 antibodies 2AC7, 9AD4, and QA1 are as described in PMID 25132548.<br>CyRPA mAbs Cy.002, Cy.003, Cy.004, Cy.005, Cy.007, Cy.009, and Cy.010 are as described in PMID 35177602.<br>RIPR mAbs RP.003, RP.004, RP.006, RP.012, RP.013, RP.016, RP.017, and RP.021 were generated in this article.
Validation	Commerical secondary antibodies were validated by suppliers.<br>Rabbit-anti-RIPR and anti-CyRPA (polyclonal, primary antibody) were vaildated by ELISA and by growth-inhibition assay in this study.<br>Anti-RH5 antibodies R5.001, R5.002, R5.003, R5.004, R5.006, R5.007, R5.008, R5.009, R5.010, R5.011, R5.013, R5.014, R5.015, R5.015, R5.017, R5.017, R5.018, and R5.019 were validated in PMID 31204103 and PMID 25132548.<br>CyRPA mAbs Cy.002, Cy.003, Cy.004, Cy.005, Cy.007, Cy.009, and Cy.010 were validated in PMID 35177602.<br>RIPR mAbs were validated by ELISA, dot blot, and growth-inhibition assay.

Eukaryotic cell lines

Policy information about cell lines and Sex and Gender in Research

Cell line source(s)	Commercial Expi293 cells (Thermo Fisher), Sf9 (Oxford Expression Technologies), and S2 cells (ExpreS2ion Biotechnologies) were used for recombinant protein expression and are commercially available. Plasmodium cell lines are derived from P. falciparum 3D7 clone originating from Dr David Walker, Edinburgh University.
Authentication	Cell lines were not authenticated except for recombinant expression of the target protein.
Mycoplasma contamination	All cell lines (Plasmodium and those used for recombinant expression) were not tested for mycoplasma contaminiation.
Commonly misidentified lines (See ICLAC register)	3D7 is the routinely used Plasmodium falciparum laboratory strain for which the first whole genome sequence was available; it is not listed in ICLAC.

Animals and other research organisms

Policy information about studies involving animals; ARRIVE guidelines recommended for reporting animal research, and Sex and Gender in Research

Laboratory animals	Female 16-week-old New Zealand white rabbits.<br>Female 8-12-week-old Wistar rats.<br>Female 6-week-old BALB/c mice.<br>Female 6-week-old SJL mice.
Wild animals	No wild animals were used in this study
Reporting on sex	Female animals were used in all experiments.

Field-collected samples	No field collected samples were used in this study
Ethics oversight	All mouse studies were carried out in compliance with the UK Animals (Scientific Procedures) 1986 Act (ASPA) under project licence PP7770851 and approved by the University of Oxford's Animal Welfare and Ethical Review Board. Rat and rabbit immunisations were by carried out by Noble Life Sciences (Woodbine, MD, USA) which is AALACi accredited and OLAW assured, studies were approved by the University of Oxford's Animal Welfare and Ethical Review Body.<br><br>Rabbits immunisations were performed by Cambridge Research Biochemicals (CRB, Billingham, UK) in compliance with the UK Animals (Scientific Procedures) 1986 Act (ASPA) and by Noble Life Sciences (Woodbine, MD, USA, which is AALACi accredited and OLAW assured).<br><br>Rat immunisations were performed by Noble Life Sciences, Inc (Woodbine, MD, USA which is AALACi accredited and OLAW assured).

Note that full information on the approval of the study protocol must also be provided in the manuscript.

Plants

Seed stocks	N/A
Novel plant genotypes	N/A
Authentication	N/A
